# Fast Speckle Noise Suppression Algorithm in Breast Ultrasound Image Using Three-Dimensional Deep Learning

**DOI:** 10.3389/fphys.2022.880966

**Published:** 2022-04-13

**Authors:** Xiaofeng Li, Yanwei Wang, Yuanyuan Zhao, Yanbo Wei

**Affiliations:** ^1^ Department of Information Engineering, Heilongjiang International University, Harbin, China; ^2^ School of Mechanical Engineering, Harbin Institute of Petroleum, Harbin, China; ^3^ Heilongjiang Provincial Hospital, Harbin, China; ^4^ School of Automatic Control Engineering, Harbin Institute of Petroleum, Harbin, China

**Keywords:** three-dimensional deep learning, image speckle suppression, bootstrap filtering algorithm, convolutional cloud network, breast ultrasound image

## Abstract

The rapid development of ultrasound medical imaging technology has greatly broadened the scope of application of ultrasound, which has been widely used in the screening, diagnosis of breast diseases and so on. However, the presence of excessive speckle noise in breast ultrasound images can greatly reduce the image resolution and affect the observation and judgment of patients’ condition. Therefore, it is particularly important to investigate image speckle noise suppression. In the paper, we propose fast speckle noise suppression algorithm in breast ultrasound image using three-dimensional (3D) deep learning. Firstly, according to the gray value of the breast ultrasound image, the input breast ultrasound image contrast is enhanced using logarithmic and exponential transforms, and guided filter algorithm was used to enhance the details of glandular ultrasound image, and spatial high-pass filtering algorithm was used to suppress the excessive sharpening of breast ultrasound image to complete the pre-processing of breast ultrasound image and improve the image clarity; Secondly, the pre-processed breast ultrasound images were input into the 3D convolutional cloud neural network image speckle noise suppression model; Finally, the edge sensitive terms were introduced into the 3D convolutional cloud neural network to suppress the speckle noise of breast ultrasound images while retaining image edge information. The experiments demonstrate that the mean square error and false recognition rate all reduced to below 1.2% at the 100th iteration of training, and the 3D convolutional cloud neural network is well trained, and the signal-to-noise ratio of ultrasound image speckle noise suppression is greater than 60 dB, the peak signal-to-noise ratio is greater than 65 dB, the edge preservation index value exceeds the experimental threshold of 0.45, the speckle noise suppression time is low, the edge information is well preserved, and the image details are clearly visible. The speckle noise suppression time is low, the edge information is well preserved, and the image details are clearly visible, which can be applied to the field of breast ultrasound diagnosis.

## 1 Introduction

The incidence of breast disease is increasing year by year. The highest incidence of breast disease is hyperplasia of mammary glands, and breast cancer is the most common female cancer. These studies show that breast health is crucial for women, and early detection and effective treatment will greatly improve the cure rate of breast disease (Huang et al., 2019; Zhao et al., 2020). As far as the current medical diagnosis and treatment methods are concerned, the preferred medical diagnosis and treatment of breast cancer is assisted by ultrasound imaging technology. Due to its low cost and high cost performance, ultrasound imaging technology has become an important means of detecting breast cancer. Therefore, breast ultrasound imaging has also become an effective way for examination of breast health (Wang et al., 2018; Xue and He, 2021). However, ultrasound will generate a lot of speckle noises during the scattering process. Excessive speckle noise in the image will greatly reduce the image resolution, affect the doctor’s interpretation of the ultrasound image, and then hinder the observation and judgment of the patients’ condition. Therefore, it is particularly important to suppress speckle noise in breast ultrasound images, and the research on the suppression of fast speckle noise in breast ultrasound images is of great significance. As a complex machine learning method, 3D deep learning exhibits powerful functions in image sample data processing. Wang et al. (2020) proposed a three-dimensional (3D) deep learning classification method, which accurately completed data classification based on 3D deep learning based on the extraction of point features and global features. Lv et al. (2020) used deep learning for breast ultrasound image analysis and research, and deep learning computer algorithm was applied to automatically extract image features; compared with traditional methods, this method had better effect and less computation. Zhu et al. (2021) developed a scheme for automatic classification of thyroid and breast lesions in ultrasound images using Deep convolution neural network (DCNN), and proposed a general DCNN structure parameter setting with transfer learning and the same structure, trained thyroid cancer and breast cancer model separately, and tested the feasibility of this general approach with ultrasound images collected from clinical practice.

Based on the existing research results, we propose a suppression algorithm for fast speckle noise in breast ultrasound images using 3D deep learning. The main Contributions of this paper are as follows: 1) Logarithm and exponential transforms are used to enhance the contrast of the input breast ultrasound images, the details of the breast ultrasound images are enhanced by combining with the guided filtering algorithm, the over-sharpening of breast ultrasound images is suppressed using the high-pass spatial filtering algorithm, the pre-processing of breast ultrasound images is completed, and the clarity of the images is improved. 2) The introduction of 3D convolutional cloud neural network in 3D deep learning may address the feature extraction and filtering issues, which lays a solid foundation for the subsequent image speckle suppression. 3) A 3D convolutional cloud neural network is used to achieve the suppression of breast ultrasound image speckles and improve the clarity and visualization of ultrasound images, which is more conducive to the detection of breast health.

## 2 Related Works

Zhang et al. (2020) proposed the breast ultrasound image segmentation problem based on a fully convolutional network; to deal with the uncertain affiliation of pixels in fuzzy boundaries Zhang et al. (2020). converted some outputs of pixel features in AlexNet into fuzzy decision expressions, optimized the AlexNet network structure, and optimized the output of the network model using fully connected conditional random fields so as to improve the representation of spatial consistency and pixel correlation of images; however, this algorithm was inaccurate for speckle noises Jin et al. (2019). proposed a DCNN-based de-noising algorithm for breast X-ray images; the algorithm performed more detailed analysis of the original image by a convolutional cloud network constructed with different convolutional sub-networks, and trained more mature training samples for speckle noise suppression of images; the algorithm is effective in suppressing speckle noises. However, it led to easy blurring of image edges and loss of edge information Jiang et al. (2021). developed a deep learning radiomics column line graph for evaluation of breast cancer pathology after neoadjuvant chemotherapy based on pre-treatment and post-treatment ultrasound. The algorithm has good image edge preservation. However, bias adjustment for speckle noise suppression is more complex Jani et al. (2018). proposed a scatter suppression algorithm using coherent stereogram generation of images to obtain relevant information by collecting light within the scene and then separating the light, achieving sparse point aggregation and finally suppressing speckle noise; the difficulty of this algorithm is the collection of light, which has more influence on the speckle noise suppression Khan et al. (2018). proposed a medical ultrasound image speckle suppression algorithm using Schur decomposition to suppress speckle noise using resolution and edge detection. The algorithm has good speckle noise suppression effects. However, it is only applicable to coarser speckles.

The above algorithm design process focuses on its image detail enhancement method. However, it cannot retain edge information well, which leads to the degradation of breast ultrasound image clarity. To address the problems of the above algorithms, a fast suppression algorithm of breast ultrasound image speckle noise based on 3D deep learning is proposed in this study. The results show that the proposed algorithm is effectively suppress the speckle noise of breast ultrasound images, and the signal-to-noise ratio (SNR), peak signal-to-noise ratio (PSNR) and edge retention ability of the suppressed images are high, and the edge information is well preserved and the image details are clearly visible.

## 3 Methodology

### 3.1 Fast Suppression Process of Speckle Noise in Breast Ultrasound Images

**FIGURE 1:**
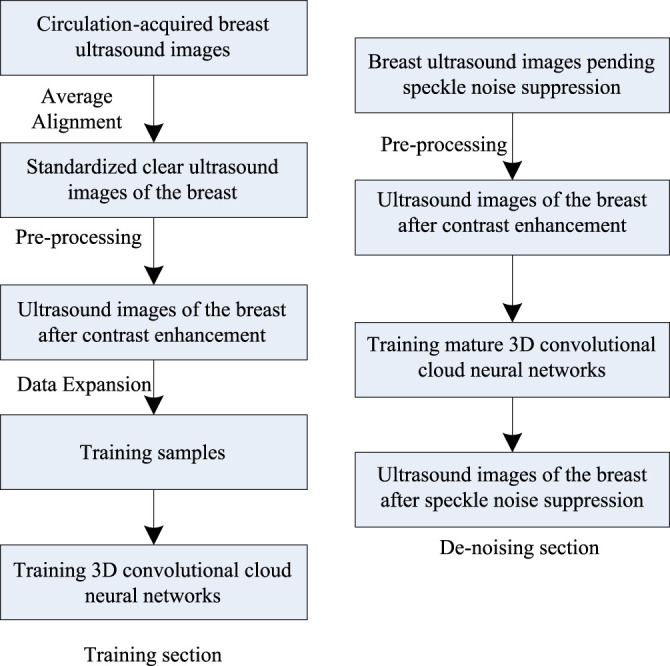
Breast ultrasound image speckle de-noising process.

Ultrasound image speckle noise suppression is usually accomplished by assuming noise as additive noise basis on the observed ultrasound image. A 3D convolutional cloud neural network with the introduction of edge-sensitive terms was applied to the breast ultrasound speckle suppression algorithm to ensure breast ultrasound image speckle noise suppression while ensuring the integrity of edge information. As shown in Figure 1.

According to Figure 1, the breast ultrasound image speckle de-noising process mainly consists of three parts: preprocessing, training and de-noising. First of all, the acquired breast ultrasound images are aligned, the standardized and clear ultrasound images are found, and the contrast increase processing is completed (Piao et al., 2018; Ren et al., 2019). After processing, data expansion processing is performed to obtain training samples, and the 3D convolutional cloud network is obtained after training on the training samples. And then, speckle de-noising is performed on the breast ultrasound images after contrast enhancement processing using the trained 3D convolutional cloud network.

### 3.2 Pre-Processing of Breast Ultrasound Image

Preprocessing of breast ultrasound images is particularly important for the suppression of ultrasound image speckle noise. The operation of preprocessing breast ultrasound images using the image-guided filter algorithm is divided into three steps. In the first step, the contrast enhancement of the input breast ultrasound image is processed. In the second step, the detail enhancement of the breast ultrasound image is achieved by the image-guided filtering algorithm. In the third step, the spatial high-pass filtering is used to suppress the over sharpening of the breast ultrasound images.

#### 3.2.1 Improving the Contrast of Input Breast Ultrasound Images

The grayscale value has an absolute effect on the clarity of breast ultrasound images (Li et al., 2020). In this study, contrast enhancement operation is performed on the input breast ultrasound images based on the grayscale values (Xu et al., 2019) using logarithmic and exponential transforms with a range of grayscale mean M from [0,260] .in which, the logarithmic transform can expand the lower gray value interval of the input breast ultrasound image f(x,y) and compress the higher gray value interval of the input breast ultrasound image f(x,y) to enhance the image contrast. The image logarithmic transformation Equation is as follows.

$$g\left(x,y\right)=\frac{b\cdot K\left(f\left(x,y\right)\right)}{c}+a$$ (1)

where a and b denote the logarithmic and exponential transformation coefficients, respectively; K(⋅) is the log-transform function; c is any constant in the interval (0,1] .

To address the phenomenon of image whitening and insufficient compensation caused by log-transforming images with too much luminance, exponential transformation was used in this study to deal with such images for effective compensation of the images (Yu et al., 2019; Tian et al., 2020). The exponential transformation Equation is as follows.

$$g\left(x,y\right)=b^{c\left(f\left(x,y\right)\right)}-b^{a}-1$$ (2)

where g(x,y) is the output image after conversion.

Combined with piecewise nonlinear transformation, we can obtain:

$$\left\{ \begin{array}{l} g\left(x,y\right)=\frac{b\cdot K\left(f\left(x,y\right)\right)}{c}+a\;0\leq M<100\\ g\left(x,y\right)=f\left(x,y\right)\;\;\;\;\;100\leq M<180\\ g\left(x,y\right)=b^{c\left(f\left(x,y\right)\right)}-b^{a}-1\;\;180\leq M<260 \end{array}\right.$$ (3)

The image grayscale value can be adjusted to a set range after transformed by Eq. 3, which in turn enhances the image contrast and facilitates better acquisition of the guide image.

#### 3.2.2 Image Enhancement Based on Image Guided Filtering

The guidance of image filtering is a transformation process, i.e., linear transformation filtering (Ji et al., 2020; Ahirwar, 2021). Therefore, assuming that the breast ultrasound image to be speckle noise suppressed with contrast enhancement completed is P , the guided image is I , and the output enhanced breast ultrasound image is Q , a weighted average sum is used to represent the filtered output $q_{i}$ of the i th pixel point of the breast ultrasound image to be speckle noise suppressed. The calculation Equation is as following.

$$q_{i}=\sum_{j}p_{j}\cdot W_{ij}\left(I\right)$$ (4)

where $p_{j}$ denotes the j -th weighted average vector, and $W_{ij}\left(I\right)$ is the filter kernel function. The calculation Equation is:

$$W_{ij}\left(I\right)=\frac{\sum_{k:\left(i,j\right)\in \omega_{k}}\left(\frac{\left(I_{i}-\mu_{k}\right)\left(I_{j}-\mu_{k}\right)}{\delta_{k}^{2}+\varepsilon }\right)}{{\left|\omega \right|}^{2}}$$ (5)

where the function leading to the image I is the kernel function $W_{ij}$ , which has no correlation with the breast ultrasound image P to be speckle noise suppressed. $\omega_{k}$ is the *i* th kernel function window, the number of pixels in the window is |ω| , the mean and variance in the window are $\mu_{k}$ and $\delta_{k}^{2}$ , respectively, and the smoothing factor is ε .

The process of image guided filtering of breast ultrasound image detail enhancement is.

$$I^{\prime }=q+EI-Eq$$ (6)

where E is the enhancement degree adjustment parameter.

#### 3.2.3 High-Pass Spatial Filtering of Breast Ultrasound Images

To achieve the goal of suppressing the components in a certain space, the spatial filter changes the image distribution frequency, thus enhancing the image contrast (Sa-Ing et al., 2018). The high-pass filter suppresses low-frequency images during local processing of image pixels to make the image clearer, which effectively avoids local over-sharpening caused by image-guided filtering processing (Lei et al., 2018).

The high-pass filter template H(r,s) is.

$$H\left(r,s\right)=\frac{1}{7}\left[\begin{array}{ccc} -1 & -2 & -1\\ -2 & 19 & -2\\ -1 & -2 & -1 \end{array}\right]$$ (7)

The output breast ultrasound image g(x,y) after image-guided filtering enhancement is:

$$g\left(x,y\right)=\sum_{r=0}^{k}f\left(x-r,y-s\right)\sum_{r=0}^{k}H\left(r,s\right)$$ (8)

where r and s denote different high-pass filter template parameters, x and y represent different breast ultrasound image parameters, and k denotes the maximum of the template parameters.

Combining Eqs 6–8, the spatial high-pass filtered breast ultrasound image $I^{\prime \prime }\left(x,y\right)$ can be obtained as:

$$I^{\prime \prime }\left(x,y\right)=\sum_{r=0}^{k}I^{\prime }\left(x-r,y-s\right)\sum_{r=0}^{k}H\left(r,s\right)$$ (9)

In summary, the pre-processing steps of the breast ultrasound image are as follows:

Step 1 The grayscale value *M* of the input breast ultrasound image *f* is calculated and find the corresponding breast ultrasound image *g* classification according to Eq. 3 to facilitate better acquisition of the bootstrap image.

Step 2 The breast ultrasound image is used after Step 1 as the guide image *I* of the guide filtering algorithm and the image after the guide filtering operation is $I^{\prime }$ .

Step 3 The output ultrasound image $I^{\prime \prime }$ is obtained by enhancing the $I^{\prime }$ edge information with high-pass filtering to maximize the retention of edge information.

### 3.3 Image Speckle Noise Suppression Model

Deem the problem of suppression of speckle noise in breast ultrasound images as an image and image mapping problem, introduce a 3D convolutional cloud neural network for speckle noise suppression, and accomplish by implementing a model training between endpoints on training samples composed of pre-processed completed breast ultrasound images.

While helping the speckle noise suppression of breast ultrasound images, the introduction of 3D convolutional cloud neural network can also cause will also cause the blurring of the edge of breast ultrasound image and the loss of information in the process of speckle noise suppression. Therefore, based on the introduction of 3D convolutional cloud neural network, the proposed algorithm takes the explicit loss of edge information as the objective of the modification function, so as to minimize the probability of losing the edge information of breast ultrasound images during the speckle noise suppression.

#### 3.3.1 3D Convolutional Cloud Neural Network Architecture

**FIGURE 2:**
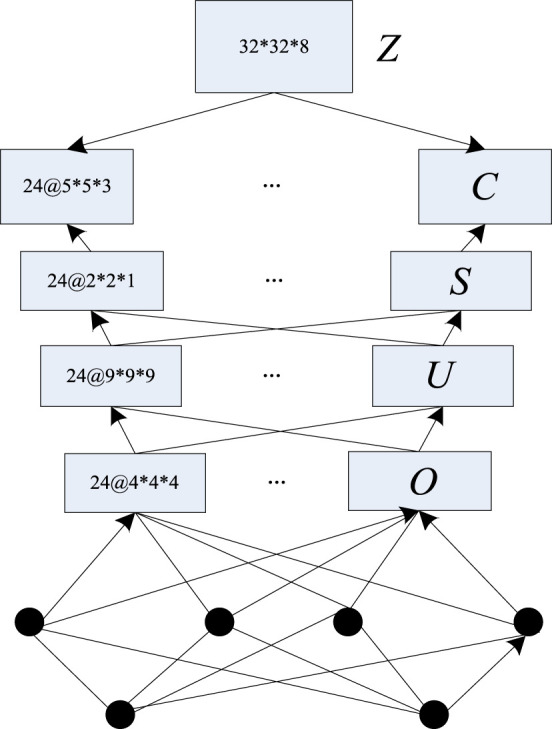
3D convolutional cloud neural network architecture.

The preprocessed breast ultrasound images were taken as the input of the 3D convolutional cloud neural network model, and the output of the model was the breast ultrasound images after speckle noise suppression. The structure for 3D convolutional cloud neural network is divided into five parts: input layer, convolution layer, pooling layer, full-connected layer and output layer. The specific 3D convolutional cloud neural network architecture is shown in Figure 2_._

In Figure 2, Z , C , S , U and O denote the input layer, the convolution layer, the pooling layer, the full-connected layer, and the output layer, respectively and their sizes are 32*32*8, *35*5*3, 2*2*1, 9*9*9, and 4*4*4, respectively. in which, the convolution layer and the pooling layer are set in rotation, the full-connected layer is set before the output layer, and the input and output _data_ of the convolution layer and the pooling layer are all 3D feature bodies. First, the 3D convolution kernels of all the convolution layers are determined so that the stacked high-level features can be selected, a new feature space can be generated using the 3D convolution kernels of different convolution layers, and then the nonlinear function can be activated by increasing the bias term (AbouJaoude et al., 2020).

The most important part in the convolutional cloud neural network is the pooling layer, which can reduce the amount of features without changing the local information of the features. Setting the convolution layer as l , then the pooling layer is $T=\left[h_{1}^{l},h_{2}^{l},h_{3}^{l},h_{4}^{l},...h_{k}^{l}\right]\in R^{X\times Y\times Z\times K}$ , and the input of the convolution layer is $T=\left[h_{1}^{l},h_{2}^{l},h_{3}^{l},h_{4}^{l},...h_{k}^{l}\right]\in R^{X\times Y\times Z\times K}$ . The operation of the maximum pooling layer needs to select the maximum value of the cube of the feature amount, which in turn generates the output $T^{\prime }\in R^{X^{\prime }\times Y^{\prime }\times Z^{\prime }\times K^{\prime }}$ . For maximum pooling of features, the feature space size before extraction is (X,Y,Z) , the features of size after extraction is $\left(X^{\prime },Y^{\prime },Z^{\prime }\right)$ , and the number of spaces of features is *K*.

Within the full-connected layer, all the nerves are connected to the neurons in the adjacent layer.

The 3D convolutional cloud sample collection composed of breast ultrasound images obtained after the above preprocessing is used as the input of the 3D convolutional cloud neural network based image speckle noise suppression model for model training, and the output of the model output layer is the breast ultrasound images after speckle noise suppression.

#### 3.3.2 Fast Speckle Noise Suppression Algorithm in Breast Ultrasound Image

The steps of the suppression algorithm for fast speckle noise in breast ultrasound images based on 3D deep learning are as follows:

Input: Original breast ultrasound image f(x,y) is preprocessed and a 3D convolutional cloud neural network architecture is constructed.

Output: Breast ultrasound images after speckle noise suppression.

Step 1According to the grayscale value of breast ultrasound images, use logarithm and exponential transforms to enhance the contrast of the input breast ultrasound images, combine with the guided filter algorithm to enhance the details of the glandular ultrasound images, adopt the spatial high-pass filtering algorithm to suppress the over-sharpening of the breast ultrasound images, complete the pre-processing of the breast ultrasound images and improve the clarity of the images.

Step 2Construct a 3D convolutional cloud neural network and input the preprocessed breast ultrasound images into the speckle noise suppression model of 3D convolutional cloud neural network images.

Step 3In the process of speckle noise suppression of breast ultrasound images, it is prone to the loss of image edge information. Therefore, the loss of edge information needs to be clarified when processing the edge information, so as to achieve intact retention of speckle noise suppression edge information. Edge information loss definition.

$$L_{Edge}\left(G\right)=\text{l}\text{o}\text{g}\frac{I^{\prime \prime }\left(x,y\right)}{\sum_{i,j}\left|y_{i+1,j}-y_{i,j}\right|}$$ (10)

where i and j denote the pixel level and vertical coordinates of images; $y_{i,j}$ denotes the edge retention coefficient of an image with image pixel level i and vertical coordinate j ; $y_{i+1,j}$ denotes the edge retention coefficient of an image with pixel level i+1 and vertical coordinate j .

Step 4Based on the analysis of the above steps, the edge similarity between canonical clear images of breast ultrasound images is measured using edge loss pairs to calculate ultrasound image gradients. The specificity of the breast structure makes the gradient in the vertical direction more important. Therefore, breast ultrasound images are represented using first-order difference in the vertical direction. The objective function obtained by integrating the edge loss $L_{Edge}\left(G\right)$ and $L_{1}$ distance with the 3D CNN is:

$$\begin{array}{l} G^{*},D^{*}=\arg\underset{G}{\min}\underset{D}{\max}L_{3DCNN}\left(G,D\right)\\ +aL_{L_{1}}\left(G\right)+\beta L_{Edge}\left(G\right) \end{array}$$ (11)

where $L_{3DCNN}\left(G,D\right)$ and $L_{L_{1}}\left(G\right)$ denote the loss functions of 3D convolutional clouds and distances, respectively. The $L_{1}$ distance term is α and the weighting factor of the edge loss is β . To ensure the stability and convergence speed of the 3D convolutional cloud neural network training, the number of $L_{1}$ distance and edge loss terms should be kept similar.

Step 5In the training of the model by the 3D convolutional cloud neural network, add the loss function to optimize the edge detail enhancement aspect of the ultrasound images, so that the completed model is more sensitive in ultrasound image edge speckle noise suppression and the speckle noise suppression effect of breast ultrasound images can be improved.

Step 6Iteratively run the convolutional neural network algorithm to determine whether the convergence condition is satisfied; if not, assuming t=t+1 , return to step 4) until convergence of the algorithm ends.

Step 7Through the operation of edge-sensitive terms in the 3D convolutional cloud neural network through the above steps, the edge information of the image is kept intact while suppressing the speckle noise of the breast ultrasound images.

Step 8End.

**FIGURE 3:**
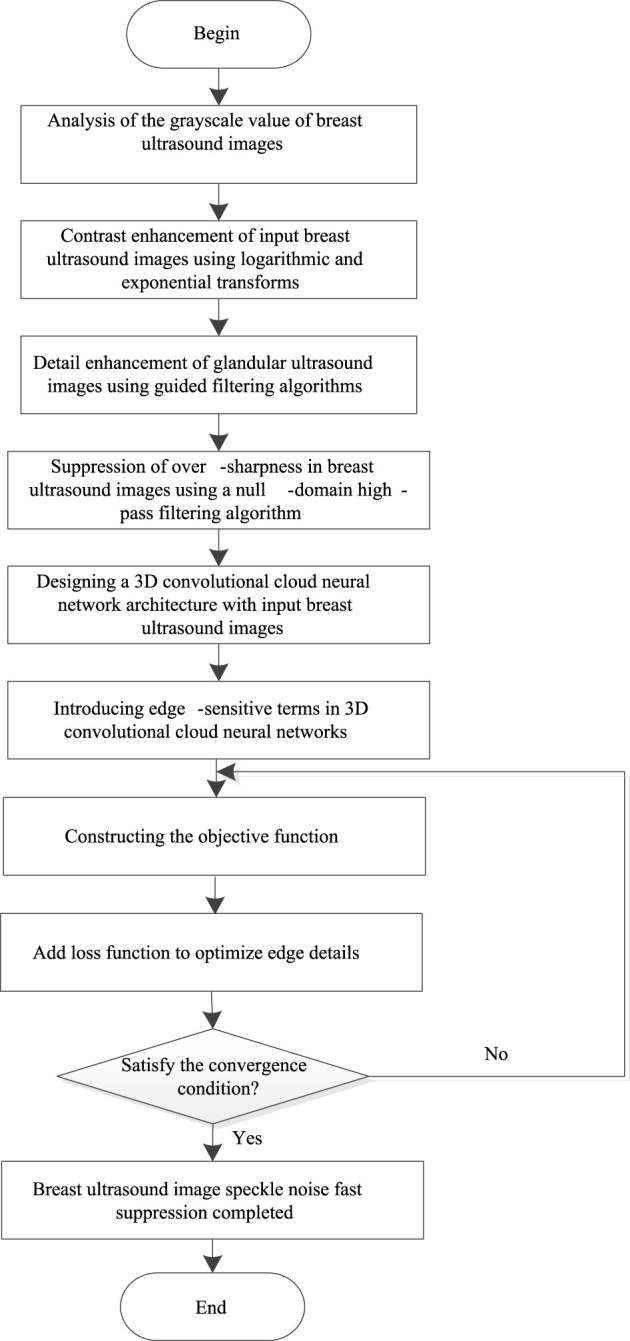
Fast speckle noise suppression algorithm process in breast ultrasound image.

Based on the above analysis, the process of the fast speckle noise suppression algorithm in breast ultrasound image using 3D deep learning is shown in Figure 3.

## 4 Experimental Analysis and Results

### 4.1 Data Sets

The experimental data was obtained from the INbreast dataset and, the DDSM data set. INbreast data set: A mammographic database with images obtained from the Breast Cancer Center located at the University Hospital (Breast Cancer Center, São João Hospital, Porto, Portugal). INbreast had 115 cases (410 images), 90 of which were from women with both breasts (4 images each), while 25 were from mastectomy patients (2 images each). Several types of lesions were included (masses, calcifications, asymmetries and deformities). The specialist also provided the precise contours in XML format. DDSM data set: One of the more well-known and voluminous data sets in the breast data set is divided into four subfolders. One of the more famous and large data sets in the breast dataset is divided into four subfolders: benign_without_callbacks, benigns, cancers and normal, respectively. They represent different categories of breast examinations, and each subfolder has many cases, each case represents a sample. In the above two datasets, one thousand breast ultrasound images are selected and speckle noise is quickly suppressed using the proposed algorithm in this paper. where, eight hundred images are randomly selected as training samples and input into the 3D convolutional cloud neural network for training, and the remaining two hundred images are used as test samples for analysis.

### 4.2 Evaluation Criteria

The algorithms of Zhang et al. (2020), Jin et al. (2019), Jiang et al. (2021), Jani et al. (2018), and Khan et al. (2018) are selected to compare and analyze the performance of speckle suppression with the proposed algorithm.

The specific Evaluation criteria are as follows:

1) The training effect of 3D convolutional cloud neural network: The lower the mean square error (MSE) and false recognition rate in suppressing the speckle noise recognition of breast ultrasound images, the better the training effect.
2) Speckle noise suppression effect of different breast ultrasound images: The closer the PSNR and SNR of the suppressed breast ultrasound images are to the threshold values of 50 dB and 65 dB of both, the better the noise suppression effect is. The PSNR is calculated as follows.

$$PSNR=10\cdot \log_{10}\left(\frac{\max_{I}^{2}}{MSE}\right)$$ (12)

where $\max_{I}$ denotes the maximum value of the image color points, and MSE denotes the image mean square deviation.

3) The edge preservation index (EPI) results for speckle noise suppression of breast ultrasound images: In addition to SNR and PSNR, the retention of image edge information after suppression is also an important evaluation index for the evaluation of the ultrasound image speckle noise suppression algorithm of the proposed algorithm. The magnitude of the EPI represents the strength of the edge retention performance; the larger the value, the stronger the EPI is. The Equation for the experimental measurement of EPI is as follows:

$$EPI=\frac{\sum \left(\Delta x-\bar{\Delta }x\right)}{\sqrt{{\sum \left(\Delta x-\bar{\Delta }x\right)}^{2}}}\frac{\sum \left(\Delta y-\bar{\Delta }y\right)}{\sqrt{{\sum \left(\Delta y-\bar{\Delta }y\right)}^{2}}}$$ (13)

where x is the speckle noise suppression of the image to be ultrasounded, y is the ultrasound image after speckle noise suppression, and Δ is the Laplace operator.

4) Speckle noise suppression quality of breast ultrasound images: The higher the image quality of breast ultrasound images after speckle noise suppression, the better the suppression effect.
5) Speckle noise suppression effect of breast ultrasound images: The shorter the time of speckle noise suppression after using different methods, the better the effect. The suppression time is calculated as follows:

$$T=\sum_{i=1}^{n}t_{i}$$ (14)

### 4.3 Results and Discussion

**FIGURE 4:**
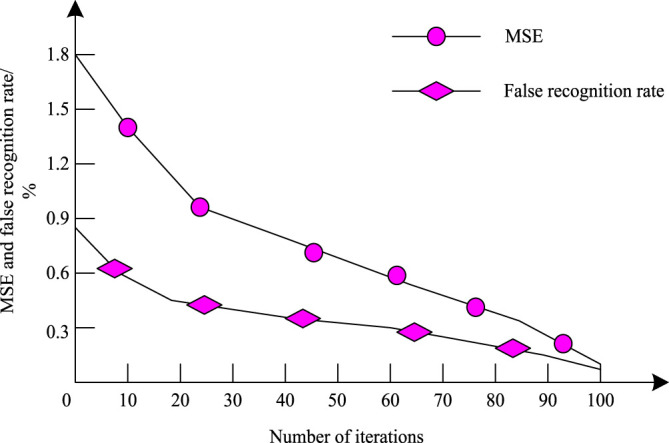
3D convolutional cloud neural network training results.

To verify the training results of the proposed algorithm using the 3D convolutional cloud neural network on the samples, the authors took the two data sets of breast ultrasound images as the object, adjusted the experimental image to the ultrasound image of pixels, used it as the input images of the 3D convolutional cloud neural network for iterative experiments, and observed the relationship between the MSE and the false recognition rate of image speckle noise recognition by different number of iterations. As shown in Figure 4.

According to Figure 4, The training MSE of the 3D convolutional cloud neural network used in the proposed algorithm is consistently higher than the false recognition rate. Both the MSE and the false recognition rate are decreasing as the number of iterations increases. At the 100th iteration, both the MSE and the false recognition rate drop to below 1.2%. This indicates that the convergence performance of the MSE and false recognition rate of image speckle noise recognition is good, the error rate is low, and the 3D convolutional cloud neural network is better for ultrasound image training.

**FIGURE 5:**
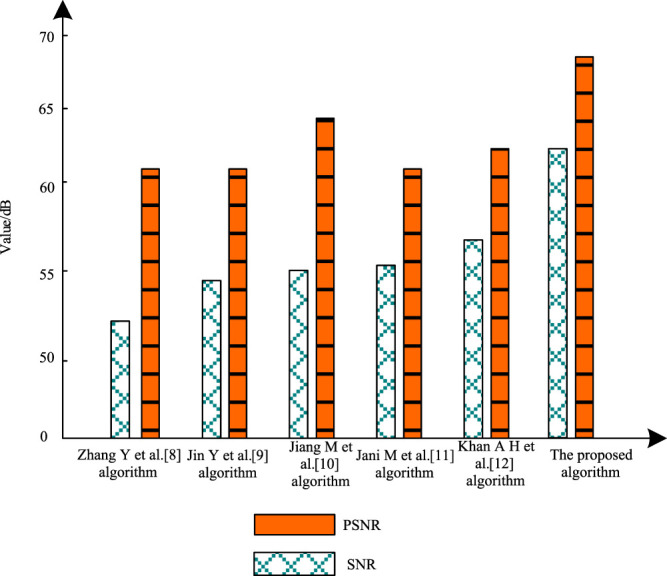
SNR and PSNR results after speckle noise suppression.

In the experiment, a representative set of breast ultrasound images was randomly selected from the two data sets as the experimental object, and the speckle noise suppression experiments were performed using the proposed algorithm to test the SNR and peak SNR. Besides, the expected SNR and peak ratio thresholds of the speckle noise suppressed ultrasound images were set to 50 dB and 65 dB, respectively. As shown in Figure 5.

According to Figure 5, after the proposed algorithm processed breast ultrasound images, the SNR was greater than 60 dB and the peak SNR was greater than 65 dB after speckle noise suppression of ultrasound images, and both SNR and peak SNR were higher than the experimental thresholds. In contrast, although the SNR of ultrasound images using the algorithms of Zhang et al. (2020), Jin et al. (2019), Jiang et al. (2021), Jani et al. (2018), and Khan et al. (2018) after the speckle noise suppression is greater than the threshold of 50 dB, it is much lower than the proposed algorithm, and the peak SNR after ultrasound image speckle noise suppression using the proposed algorithm does not reach 65 dB using the algorithms of Zhang et al. (2020), Jin et al. (2019), Jiang et al. (2021), Jani et al. (2018), and Khan et al. (2018). This illustrates the good effect of the proposed algorithm on breast ultrasound image speckle noise suppression.

**FIGURE 6:**
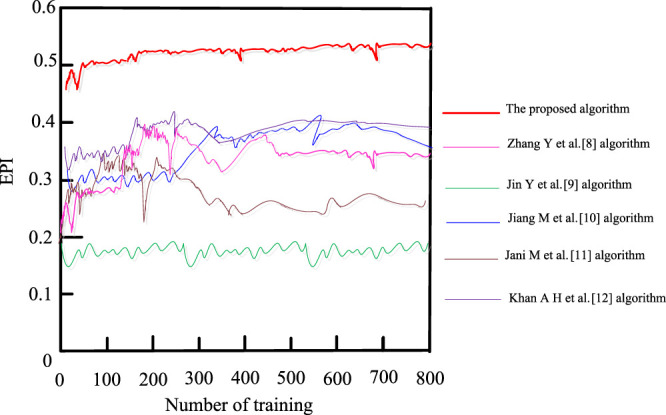
EPI results after speckle noise suppression.

In the experiment, the EPI threshold was set to 0.45 and the statistics on EPI of breast ultrasound images after suppression were performed using the proposed algorithm. As shown in Figure 6.

It is seen from Figure 6 that the EPI values of the suppressed ultrasound images using the proposed algorithm were all high and exceeded the experimental threshold of 0.45 Zhang et al. (2020) and Jiang et al. (2021) and Khan et al. (2018) algorithms have relatively high EPI values for the suppressed ultrasound images, reaching 0.40 but not higher than 0.45. The Jani et al. (2018) algorithm has a weaker EPI for ultrasound images with the highest EPI value of 0.33 only, and the Jin et al. (2019) algorithm has the lowest value, always below 0.2. This indicates that the proposed algorithm does not cause loss of edge information while speckle noise is suppressed, resulting in good visualization of breast ultrasound images.

**FIGURE 7:**
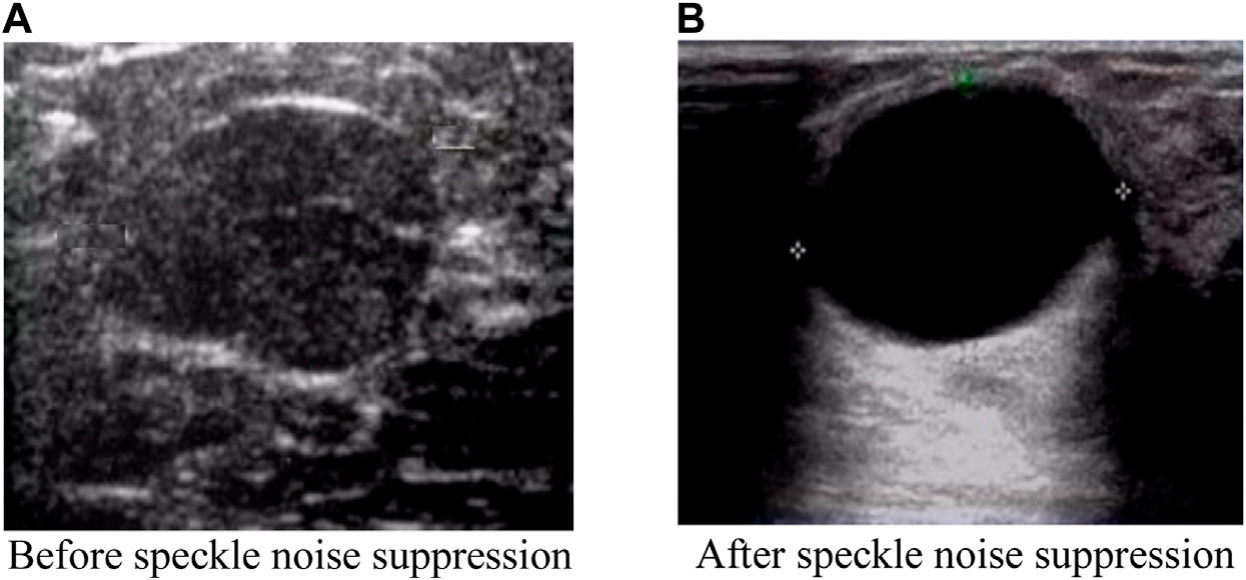
Comparison effect after image speckle suppression (INbreast dataset). **(A)** Before speckle noise suppression **(B)** After speckle noise suppression.

**FIGURE 8:**
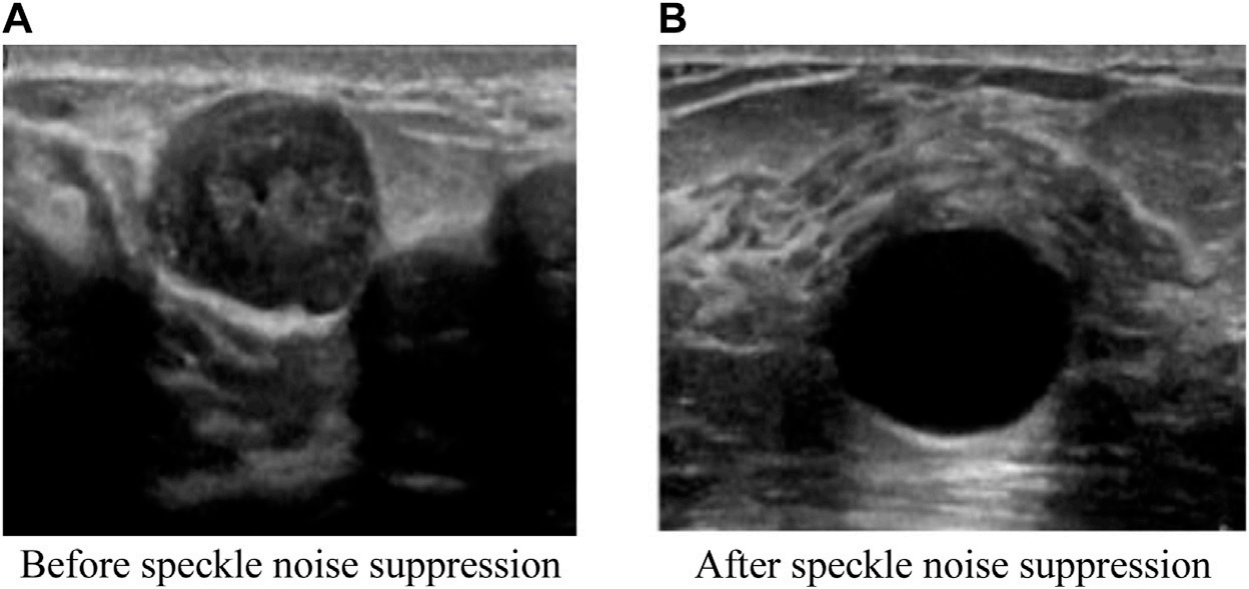
Comparison effect after image speckle suppression (DDSM dataset). **(A)** Before speckle noise suppression **(B)** After speckle noise suppression.

To observe the intuitive effect of the proposed algorithm on the suppression of ultrasound image speckle noise, a breast ultrasound image was selected from the INbreast dataset and the DDSM dataset respectively, and the proposed algorithm was used to suppress the speckle noise on the breast ultrasound image. The comparison of before and after image speckle noise suppression is shown in Figures 7, 8.

It can be seen from Figures 7, 8 that the original breast ultrasound images selected from both datasets contained a large number of speckles, which resulted in inaccurate identification of the breast health status and easy overlooking of small lesions. However, after the speckle noise suppression using the proposed algorithm, the details of the ultrasound images become regular and clear, and the images are smooth and uniformly distributed. In addition, the proposed algorithm has the effect of good retention of edge information and clear visibility of details after ultrasound image refinement, which facilitate better observation of the breast health status. This shows that the proposed algorithm is effectively suppress the speckle noise of breast ultrasound images while retaining the information well.

To verify the speckle noise suppression effect of the proposed algorithm, the proposed algorithm is experimentally compared with the algorithms in Zhang et al. (2020), Jin et al. (2019), Jiang et al. (2021), Jani et al. (2018) and Khan et al. (2018) for a cross-sectional comparison of the speckle noise suppression effects. The de-noising results of the three algorithms were compared by adding white Gaussian noise of 20 dB, 40 dB, and 60 dB to the breast ultrasound images of INbreast dataset and DDSM dataset, respectively, using PSNR and de-noising time as indicators. The experimental results are shown in Table 1.

**TABLE 1:** Comparison of speckle noise suppression effects of different algorithms.

White Gaussian noise/dB	INbreast data set			DDSM data set		
	20 dB	40 dB	60 dB	20 dB	40 dB	60 dB
The proposed algorithm	8	10	11	13	17	15
Zhang et al. (2020) algorithm	23	21	26	24	26	20
Jin et al. (2019) algorithm	12	15	18	16	18	21
Jiang et al. (2021) algorithm	19	23	26	18	19	25
Jani et al. (2018) algorithm	12	23	25	20	22	21
Khan et al. (2018) algorithm	21	23	25	18	22	25

According to the data in Table 1, after adding different amounts of white Gaussian noise to the INbreast dataset and the DDSM dataset, the proposed algorithm has an overall lower noise than the other algorithms, and the highest noise is significantly smaller than the other algorithms. The highest noise of the proposed algorithm is 17 dB, whereas the highest noise of the algorithms in Zhang et al. (2020) and Jiang et al. (2021) is 26 dB, the highest noise of the algorithm in Jin et al. (2019) is 21 dB, and the highest noise of the algorithms in Jani et al. (2018) and Khan et al. (2018) is 25 dB. This shows that the proposed algorithm has less sensitivity to noises and higher efficiency of speckle suppression.

## 5 Conclusions and Future Works

The effective processing and application of 3D breast ultrasound images can largely improve the objectivity and manipulability of clinical diagnosis. To ensure the quality of breast ultrasound images and avoid the adverse effects of noise interference, this study uses 3D convolutional cloud neural network, guided filter algorithm and high-pass spatial filtering algorithm to achieve the speckle noise suppression of ultrasound images. It is proved in the experiment that the proposed algorithm can effectively suppress the speckle noise of breast ultrasound images, and the edge information is well retained and the image details are clearly visible, which provides a certain foundation for the intelligent processing and application of breast ultrasound images. However, the noise features and other related features of breast ultrasound images are not deeply examined in this study. In future works, it is expected to explore more detailed information about the features of breast ultrasound images, so as to further improve the understanding of breast ultrasound images and facilitate better application of ultrasound images in clinical treatment.

## Data Availability

The original contributions presented in the study are included in the article/Supplementary Material, further inquiries can be directed to the corresponding author.
